# Percutaneous Transforaminal Full-Endoscopic Removal of Neurinoma of the Fifth Lumbar Nerve Root With Intraoperative Neuromonitoring: A Case Report

**DOI:** 10.3389/fsurg.2022.877974

**Published:** 2022-04-29

**Authors:** Maxim N. Kravtsov, Vadim A. Manukovsky, Saidmirze D. Mirzametov, Olga V. Malysheva, Dmitry A. Averyanov, Dmitry V. Svistov

**Affiliations:** ^1^Kirov Military Medical Academy, St. Petersburg, Russia; ^2^Saint-Petersburg I.I. Dzhanelidze Research Institute of Emergency Medicine, St. Petersburg, Russia; ^3^North-Western State Medical University Named After I.I. Mechnikov, St. Petersburg, Russia

**Keywords:** percutaneous endoscopic surgery, full-endoscopic spine surgery, transforaminal approach, spinal oncology, intraforaminal neurinoma

## Abstract

**Background:**

Technical achievements and surgical techniques improvement contribute to the expansion of the endoscopic spine surgery possibilities. However, today there are few reports about the use of percutaneous endoscopy in spinal tumor surgery. A case of percutaneous transforaminal endoscopic removal of the lumbar spinal nerve tumor with intraoperative neuromonitoring is presented.

**Case Description:**

A 59-year-old female was complaining of a left shin and foot pain, weakness, and paresthesia. Preoperative magnetic resonance imaging (MRI) revealed a tumor (neurinoma) at the left L5-S1 intervertebral foramen. Transforaminal endoscopic removal of an extramedullary tumor from an 8-mm skin incision with intraoperative neuromonitoring was performed. Postoperative MRI revealed the signs of total resection of the tumor.

**Conclusion:**

The presented case confirms that percutaneous endoscopic removal of lumbar spine intraforaminal neurinomas can be safe and effective.

## Introduction

Percutaneous full-endoscopic spine surgery is known for over 30 years (1). However, only in the 2000s, it became popular in clinical practice thanks to development of the surgery technique of a percutaneous endoscopic access to the spinal canal and clear visualization of neural structures (2–4). During that period, the approach changed from spinal arthroscopy (discoscopy) to spinal neuroendoscopy. It triggered a fast improvement of the technique itself and upgrade of surgical instruments for percutaneous endoscopy of the spine, thereby determining new indications for this type of surgery. Meeting all criteria for minimally invasive surgery, percutaneous endoscopic interventions are of great interest to specialists and in demand by patients (5).

Main indication for percutaneous endoscopic intervention on the spine is degenerative–dystrophic pathologies (6). Apart from that, this technique has been tried in infectious spine diseases (7, 8); chronic epidural hematoma (9); and spine stabilization and its complications (10–12).

In 2012, first reports were published on percutaneous endoscopy for extradural neoplasms of the spine (13, 14), and in 2019 for removal of intradural extramedullary tumors (15). However, the surgical technique, safety, and efficiency of percutaneous endoscopy for spine tumors have not been sufficiently described in the publications. This paper presents a case report of full-endoscopic transforaminal removal of lumbar neurinoma with intraoperative neurophysiological monitoring.

### Clinical Case

**Figure 1:**
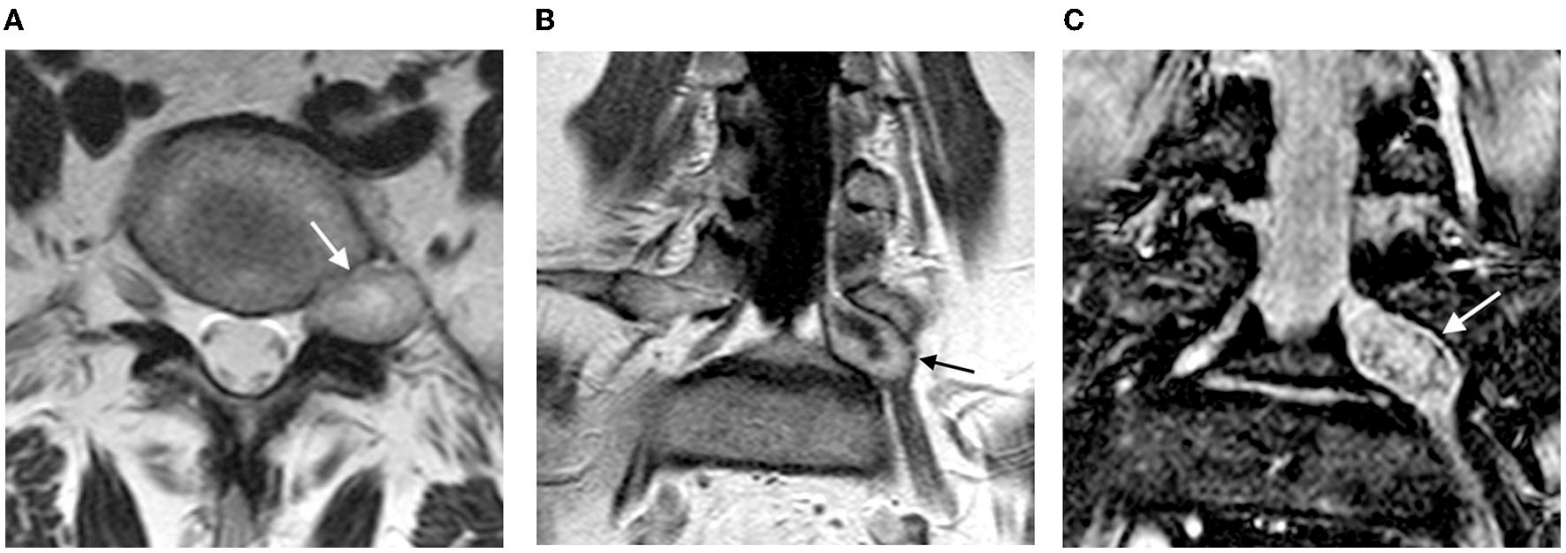
Magnetic resonance imaging of the lumbar spine: **(A)** axial view, T2-WI mode; **(B)** frontal view, T1-WI contrast mode; **(C)** frontal view, T1-FS post-contrast (arrow shows a cystic-solid tumor of the spinal nerve, size 3.2 × 1.5 × 1.5 cm; the tumor accumulates contrast).

A 59-year-old woman admitted to our clinic, with constant left leg pain lasting for 2 years. Over the past 6 months, there was a gradual increase in pain intensity up to 7–8 Visual analogue scale (VAS) scores. Magnetic resonance imaging (MRI) showed a tumor of the left L5 spinal nerve at the level of the intervertebral foramen (Figures 1A–C).

Neurological status: moderate paresis of the left foot extensors (3 points), Lasègue's sign on the left, throbbing pain and paresthesia in the L5 dermatome on the left, and no negative sensitive signs.

Electroneuromyography (ENMG) showed decreased amplitudes of motor responses on the left in abduction of *m. extensor digitorum brevis* by 30% compared to the right side. As to *mm. peroneus longus, tibialis anterior* on the left, no spontaneous activity was detected, motor unit potentials were not changed, and the interference pattern was complete. The ENMG data match a mild axonal preganglionic lesion at the L5 level on the left.

We decided to perform percutaneous full-endoscopic resection of the L5 spinal nerve tumor using a left-sided foraminal approach.

### Anesthesia and Neurophysiological Monitoring

The patient received total intravenous anesthesia with propofol and fentanyl. Muscle relaxants were used only for tracheal intubation.

Intraoperative neurophysiologic monitoring included spontaneous electromyography (free-run EMG) and monopolar direct nerves stimulation (NIM 3.0, Medtronic, Minneapolis, MN, USA). Motor-evoked potentials were recorded with needle electrodes within target muscles located by the anatomical myotomes [*mm. extensor digitorum brevis* (L5), *tibialis anterior* (L5), and *gastrocnemius* (S1) on the left]. Filter setting was made as follows: low-pass filter 30 Hz and high-pass filter 3,000 Hz. We used monopolar continuous cathode rhythmic stimulation with rectangular 4-Hz impulses, stimulus time 0.1 ms, and stimulus intensity ranging from 1.0 to 2.0 mA. Cathode monopolar stimulation was made with a modified elongated probe (based on Medtronic probe, USA) through the working channel of the endoscope. A standard needle electrode was placed at the edge of the surgical wound as reference. Monopolar stimulation was made during surgical intervention in order to assess the L5 nerve and its conductivity. Direct nerve stimulation during tumor removal at intensity 1.0 mA evoked motor potentials of the target myotome muscles of the L5 motor root. During the tumor removal no parameters of the recorded motor response significantly varied. Spontaneous electromyography at the tumor removal stage recorded patterns of minimal mechanical impact like single-spike waves in *mm. tibialis anterior, extensor digitorum brevis*.

## Surgery

**Figure 2:**
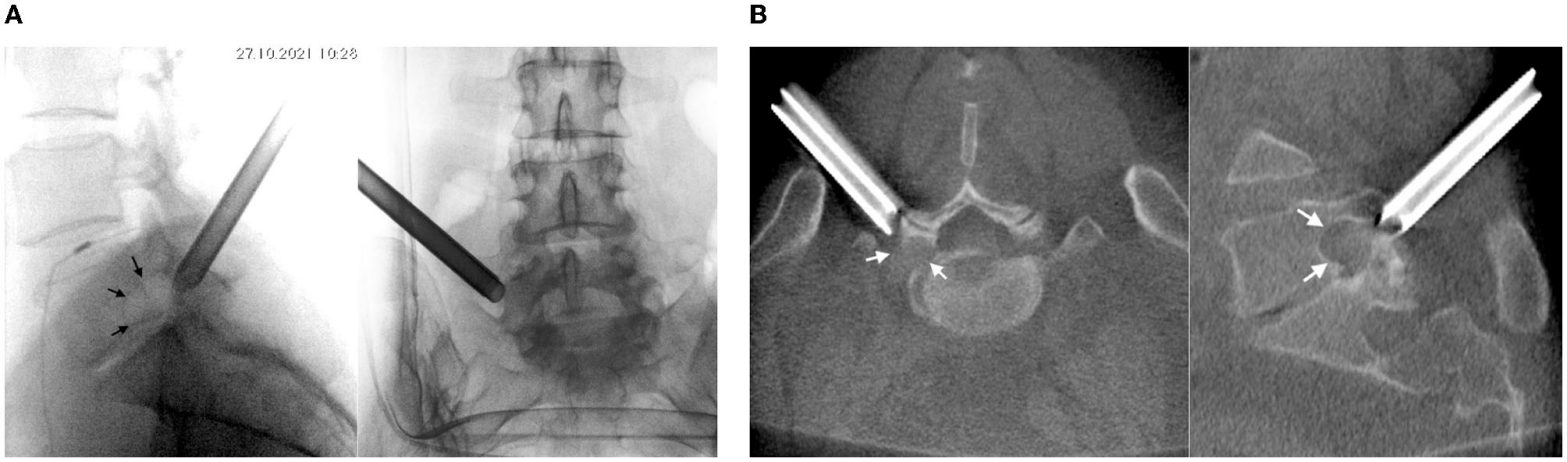
Intraoperative **(A)** X-rays and **(B)** CT (O-arm) of the position of the working tube at the left intervertebral foramen L5-S1 (see arrows).

On 27 October 2021, surgery was performed with the patient in the prone position. Guided by fluoroscopy, a puncture needle 18G was placed to the intervertebral joint L5-S1 through a point located 10 cm to the left of the midline. A guide pin was inserted along the needle, and the needle was removed. A linear cut 8-mm long was made. A soft tissue retractor was introduced into the wound along the guide pin. A working tube with diameter 7 mm was placed along the retractor, after which the pin and the retractor were removed (Figures 2A,B).

**Figure 3:**
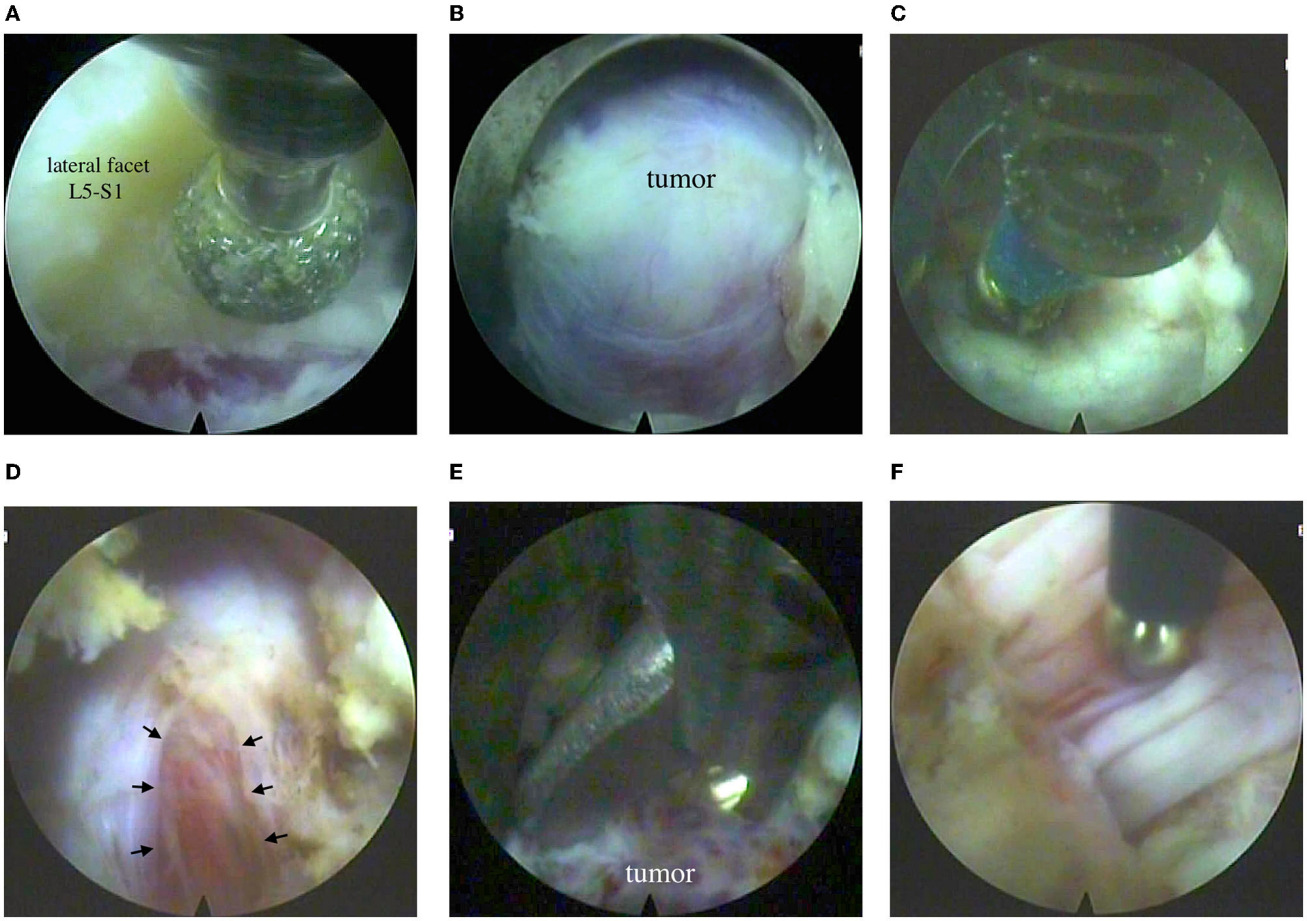
Endoscopic step of the surgery: **(A)** partial resection of the L5-S1 lateral facet on the left with a burr; **(B)** view of the tumor in the intervertebral foramen; **(C)** bipolar electrocoagulation of the nerve sheath; **(D)** incision of the nerve sheath (see arrow); **(E)** removal of the tumor with forceps; **(F)** monopolar stimulation of the nerve bundles with a modified elongated probe (based on Medtronic probe, USA).

TESSYS, Joimax® (Germany) endoscope was inserted into the working tube. Further manipulations were controlled by video endoscopy under continuous irrigation with normal saline solution. The intervertebral joint L5-S1 was‘ visualized. A partial lateral facetectomy was performed with a high-speed burr (Figure 3A). An expansive growth of the tumor resulted in enlarged intervertebral foramen, so there was no need in foraminoplasty. An intraforaminal tumor located inside the L5 spinal nerve was seen (Figure 3B).

After electrocoagulation on a small portion of the nerve sheath, an incision was made to see moderately vascularized tissue of the tumor, grayish-red in color, of soft consistency (Figures 3C,D). The tumor was removed, and functions of active motor nerve bundles were intact, which was confirmed by neuromonitoring (Figures 3E,F). Bleeding from the tumor vessel was controlled by bipolar coagulation. After a temporary stop of irrigation, endoscopic signs of stable hemostasis and the absence of the leakage of cerebrospinal fluid were revealed. The skin wound was sutured with 1 knotted suture. Blood loss was <30 ml; the surgery lasted for 120 min.

### Result of Pathological Test: Neurinoma (Grade I)

**Figure 4:**
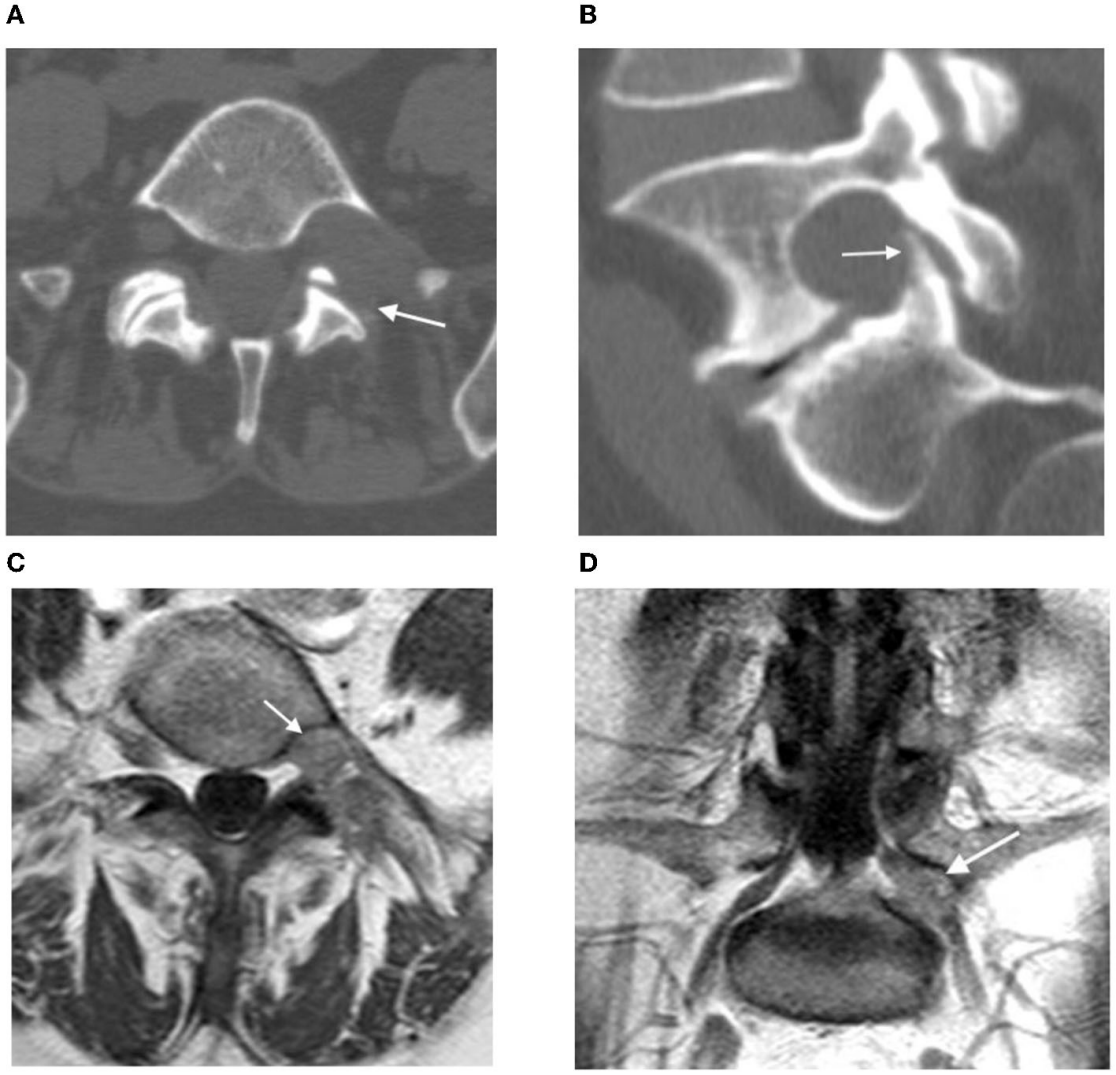
**(A,B)** Postoperative CT: the arrow shows the resection area of the left intervertebral joint L5-S1; **(C,D)** contrast-enhanced MRI 3 months after surgery: postoperative changes in the left L5 root (see arrow), no signs of contrast accumulation.

Upon discharge, the patient had a regress in severe pain of the left leg and Lasègue's sign. Postoperative CT confirmed bone resection in the extent of partial lateral facetectomy (Figures 4A,B).

The control contrast-enhanced MRI on the next day and 3 months after the surgery verified total tumor resection, with no accumulation of contrast agent (Figures 4C,D).

After 4-month follow-up, paresis of the big toe extensor on the left foot remained, up to 3 scores. Occasionally, the patient feels a slight throbbing pain in the L5 dermatome on the left (2 VAS scores).

## Discussion

The report above describes one of lumbosacral neurinoma surgical treatment methods. By Kato classifications (1993), the lesion corresponds to intraforaminal neurinoma type II (16).

There are few reports on neurinoma removal by full endoscopy. Wang et al. presented a successful percutaneous foraminal endoscopic removal of *dumbbell-shaped* neurinomas, up to 4 cm in size, in 12 patients (17). The authors suggested the following advantages of this surgery method:

1. The trajectory of transforaminal endoscopic access is optimal for localization of neurinoma.
2. Minimally invasive approach does not require significant bone resection or lead to iatrogenic spinal instability.
3. Modern advancements in transforaminal percutaneous endoscopy allow total removal of the tumor not only between the vertebral foramen, but in the spinal canal and extraforaminal area during one surgery.

We fully agree with Wang et al. in terms of the advantages of percutaneous endoscopic resection of intraforaminal neurinomas. However, we do not share their opinion that intraoperative neurophysiological monitoring is inexpedient. The authors back up their position by very rare neurological disorders after total resection of the neurinoma and consider it appropriate to transect the affected nerve completely (17).

Researchers have proved that the risk of developing a neurological deficit after removal of neurinomas and neurofibromas with complete transection of the supporting nerve can reach 23% (18), while plegia is registered in 3% of cases (19). Such conditions are caused by the rare variants of growth of motor root neurinoma (19), and by incomplete compensatory innervation of the muscles by adjacent spinal nerves roots (20). Therefore, in order to assess the risks and clarify the surgical tactics for spinal neurinomas, it is necessary to use electrophysiological control at pre- and intraoperative stages (19). In our opinion, this rule should also be applied to percutaneous endoscopic surgery.

In the case presented herein, preoperative ENMG confirmed a partially impaired conduction along the L5 root, which corresponded to the severity of neurological disorders. Intraoperative neuromonitoring with NIM3.0 system (Medtronic, USA) during percutaneous videoendoscopic resection of L5 neurinoma ensured the safety of surgical procedures, made it possible to remove tumors completely and partially retain anatomical integrity of the affected nerve, which made a positive effect on the functional outcome of treatment.

There are no previous reports on application of neurophysiological monitoring with direct monopolar nerve stimulation in percutaneous neuroendoscopic interventions on the spine (17, 21). Perhaps, it results from the lack of electrodes with the size sufficient for introducing them through the endoscope work channel. We modified a cathode monopolar stimulation probe (Medtronic, USA) by increasing its length.

Obvious obstacles to a widespread use of uniportal percutaneous endoscopic surgery for spine and spinal cord tumors today can be formulated as follows (11, 15, 17, 22, 23):

1. limited nature of methods of hemostasis and visualization of sources of bleeding;
2. lack of effective methods for sealing of the dura mater during removal of intradural neoplasms;
3. *coaxial method* of visualization and manipulation;
4. long time required for a specialist to learn the surgery technique.

Intense bleeding greatly worsens the video endoscopic image of the surgery cavity and increases the risk of complications (22). Currently known ways of hemostasis during percutaneous endoscopic removal of the spine neoplasms and spinal cord (preoperative embolization, coagulation, increased irrigation pressure, blood pressure control, etc.) are not enough (23, 24). On top of that, increased irrigation pressure after opening of the dura mater can cause complications due to intracranial hypertension (15). Therefore, percutaneous endoscopic removal of a well-vascularized tumor must be made by an experienced surgeon, otherwise preference must be given to an open intervention (24). The same principle must be applied to tumors of large size and high density (11). In our case, intraoperative bleeding was moderate, so we could use standard methods for endoscopic hemostasis.

There are different methods of sealing the dura mater in percutaneous endoscopic interventions. Conservative tactics for small defects in the dura mater, combined with hypotensive syndrome therapy, appear to be most effective (25–27). Among surgical methods to close defects in the dura mater, the most optimized are conversion to microsurgery (25), suture of the dura mater through an endoscope by Youn's technique (28), and sealing with tissue adhesive (15). In our case, extra-arachnoid localization of neurinoma did not require the dura mater plastics.

The above-listed challenges of the surgery methods can be overcome by using percutaneous unilateral biportal endoscopic technique, which has been widely developing in recent years (29). In particular, a clipping method can be good for large defects in the dura mater (27). Apart from that, percutaneous biportal endoscopy allows abandoning coaxial imaging and switching to a bimanual surgical technique, more familiar to the surgeon (30).

## Conclusion

The presented case herein shows that uniportal full-endoscopic resection of intraforaminal neurinomas of the lumbar spine with intraoperative neurophysiological monitoring is safe and effective. Further study of potential benefits and effectiveness of percutaneous endoscopic removal of spine and spinal cord tumors must involve a larger number of cases within comparative study.

## Data Availability Statement

The original contributions presented in the study are included in the article/supplementary material, further inquiries can be directed to the corresponding authors.

## Ethics Statement

Ethical review and approval was not required for the study on human participants in accordance with the local legislation and institutional requirements. The patients/participants provided their written informed consent to participate in this study.

## Author Contributions

MK, SM, OM, and DA contributed to the conception and design of the study, the analysis and interpretation of data, and the work draft. MK and SM designed figures and video. VM and DS offered guidance in study design and revised the article critically for important intellectual content. All authors read and approved the final version of the manuscript.

## Conflict of Interest

The authors declare that the research was conducted in the absence of any commercial or financial relationships that could be construed as a potential conflict of interest.

## Publisher's Note

All claims expressed in this article are solely those of the authors and do not necessarily represent those of their affiliated organizations, or those of the publisher, the editors and the reviewers. Any product that may be evaluated in this article, or claim that may be made by its manufacturer, is not guaranteed or endorsed by the publisher.
